# P-997. Congenital Cytomegalovirus and Hearing Loss and Response to Treatment with Valganciclovir

**DOI:** 10.1093/ofid/ofae631.1187

**Published:** 2025-01-29

**Authors:** Salzman B Mark, Lisa C Lin

**Affiliations:** Southern California Permanente Medical Group, Los Angeles, California; Southern California Permanente Medical Group, Los Angeles, California

## Abstract

**Background:**

Congenital cytomegalovirus (CMV) occurs in about 1 in 200 babies and is the leading non-genetic cause of sensorineural hearing loss (SNHL) in children in the United States. Approximately 50% of children with SNHL secondary to CMV have progression of their hearing loss. Data on the effectiveness of valganciclovir in the treatment of infants with congenital CMV with isolated hearing loss at birth is limited.

**Methods:**

All newborns who did not pass their newborn hearing screening at birth between 2019-2023 were included and tested for CMV by a nucleic acid test from saliva and if positive confirmed from urine. All infants with CMV detected in urine had a Brainstem Auditory Evoked Response (BAER) test by 2-3 weeks of life and if hearing loss was confirmed, offered treatment with valganciclovir for 6 months. Infants who only had CMV detected in the saliva were not included.

**Results:**

204,680 newborns received hearing screens between 2019-2023. 7147 (3.5%) did not pass their hearing screening. 509 (7.1%) had hearing loss confirmed on BAER testing. 46 of the 7147 infants who did not pass their initial hearing screen had CMV detected in saliva and 17 of those had CMV detected in urine. 5 patients never received a urine test and were lost to follow-up. Among 17 cases with confirmed congenital CMV, 9 had confirmed hearing loss, 7 had normal hearing, and one lost to follow-up. 12 of the 17 cases were treated with valganciclovir, 8 with confirmed hearing loss, 3 with normal hearing, and one unknown. Among the 8 treated with hearing loss, 3 had progression of hearing loss, one improved, one was unchanged, and 3 lost to follow-up. Among the 7 with congenital CMV and normal hearing at birth, 3 were treated with valganciclovir and 2 remain with normal hearing with one to be determined. Among the 4 not treated, 3 remain with normal hearing and one was lost to follow-up.

Among the 6638 with normal hearing on follow-up, 7-13 had congenital CMV (1 in 511-948). Among the 509 with confirmed hearing loss, there were 9 (1.8%) who had congenital CMV.

**Conclusion:**

Congenital CMV was found in only 1.8% of newborns with hearing loss and about 0.15% of newborns without hearing loss. Treatment with valganciclovir did not appear to be beneficial in preventing the progression of hearing loss in some newborns but the numbers were too small to reach a conclusion.

**Disclosures:**

**All Authors**: No reported disclosures

